# ASSESSMENT OF THE LINK BETWEEN FRAILTY AND DEMENTIA IN THE OLDEST-OLDS USING JOINT MODELS

**DOI:** 10.1093/geroni/igad104.2790

**Published:** 2023-12-21

**Authors:** Franklin Massa, Alejandra Marroig, Graciela Muniz-Terrera, Boo Johansson

**Affiliations:** Universidad de la República, Montevideo, Montevideo, Uruguay; Universidad de la Republica Uruguay, Montevideo, Montevideo, Uruguay; Ohio University, Athens, Ohio, United States; University of Gothenburg, Sweden, Gothenburg, Gothenburg, Sweden

## Abstract

Frailty and dementia affect many older adults worldwide. As the world’s population ages, the number of people experiencing frailty and dementia is increasing, posing significant challenges for individuals, health care systems and caregivers. Here, we aim to assess the link between the evolution of frailty and the time to dementia diagnosis in the oldest-old. We derived the frailty index (FI) using data from 5 waves of the OCTO-Twin Study: Origins of Variance in the old-old, and fitted joint longitudinal-survival models to repeat measures of the index and time to dementia diagnosis to understand the association between the trajectory of the FI and time to diagnosis. We accounted for possible effects of sex, education and the presence of the APOE e4 gene. A nonlinear trajectory of the frailty index (p < 0.001) and an effect of education attenuating the levels of the frailty index (p = 0.032) were observed. Additionally, the results agree with the literature regarding the effect of the APOE e4 as a greater risk for dementia (HR = 2.034, p < 0.001). Moreover, an increasing frailty trajectory is associated with an earlier time to diagnosis of dementia (HR = 1.096, p < 0.001). Our findings advance our understanding of the relation between frailty and dementia in the oldest old. Our approach accounts for the non-linearity of the trajectory of frailty and deliver a dynamic tool that updates dementia prognosis when a new measurement of frailty is available.

